# Supplementary material for: Brainstem development requires galactosylceramidase and is critical for pathogenesis in a model of Krabbe disease
Source: Nat Commun. 2020 Oct 23;11:5356. doi: 10.1038/s41467-020-19179-w (PMC7584660; doi:10.1038/s41467-020-19179-w)
Supplement: Supplementary file 1 — Supplementary Information [file 41467_2020_19179_MOESM1_ESM.pdf]

Supplementary Figures

Brainstem development requires galactosylceramidase and is critical for pathogenesis in a model of Krabbe disease

Nadav I. Weinstock[1,2], Conlan Kreher[1,2], Jacob Favret[1-3], Duc Nguyen[4], Ernesto R. Bongarzone[4], Lawrence Wrabetz[1,2,5,6], M. Laura Feltri[1,2,5,6], and Daesung Shin[1-3,6 §]

[1]Hunter James Kelly Research Institute, [2]Department of Biochemistry, [3]Department of Biotechnical and Clinical Laboratory Sciences, Jacobs School of Medicine and Biomedical Sciences, University at Buffalo (SUNY), Buffalo, NY 14214, USA. [4]Department of Anatomy and Cell Biology, College of Medicine, University of Illinois at Chicago, Chicago, IL 60612, USA. [5]Department of Neurology, [6]Neuroscience Program, Jacobs School of Medicine and Biomedical Sciences, University at Buffalo (SUNY), Buffalo, NY 14214, USA

§To whom correspondence should be addressed: Daesung Shin, 26 Cary Hall, 3435 Main street, Jacobs School of Medicine and Biomedical Sciences, University at Buffalo (SUNY), Buffalo, NY 14214, e-mail: daesungs@buffalo.edu, Tel: 716-829-5191, Fax: 716-829-3601

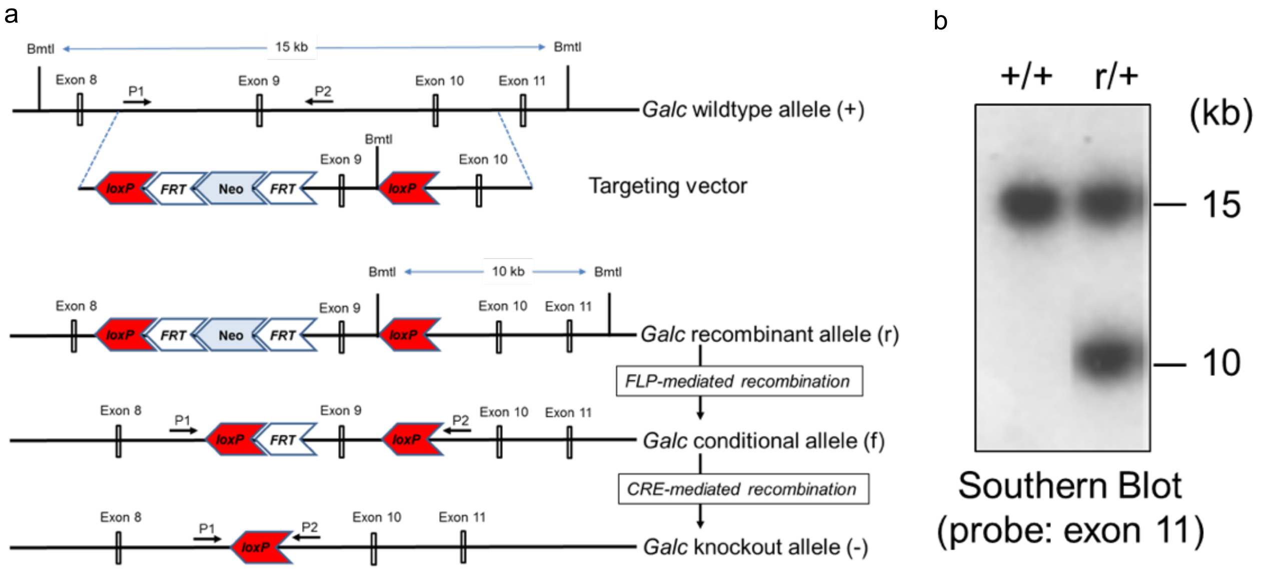

Figure S1. **Design of conditional *Galc* floxed allele mouse**. (**a**) Structure of the wild-type (+) locus, the targeting vector, the targeted recombinant allele (r), the conditional allele (f), and the deleted KO (-) *Galc* (exon 9) allele, the loxP and FRT recombination sites (red and empty, respectively), the *Bmt*I restriction enzyme sites and exons, and the P1-P2 primers for PCR genotyping are indicated. (**b**) Southern blot analysis of the wild-type (+/+) ES cells and of the recombinant (r/+) clones. *Bmt*I-restricted genomic DNA yielded 15- and 10-kb bands for the wild-type and recombinant alleles, respectively, with the probe on exon 11 of the *Galc* gene. The experiment was repeated twice with samples from different animals.

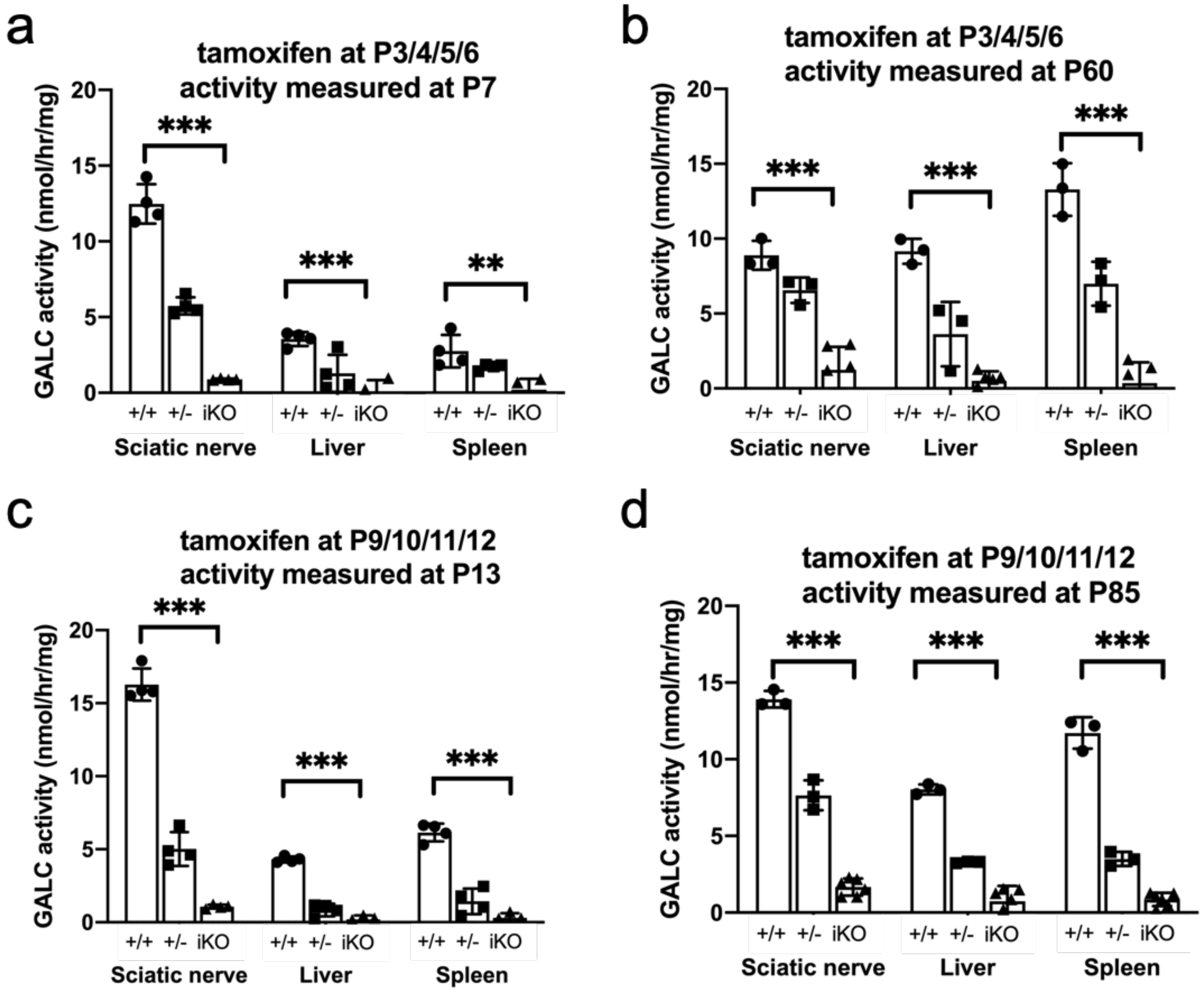

Figure S2. Residual GALC activities of peripheral tissues after *Galc*-iKO TM induction. (**a, c**) 24 hours after tamoxifen induction, peripheral tissues including sciatic nerve, liver and spleen had only 6%, 1-4% and 5-7% of WT GALC activity remaining, respectively, in both early (**a**) and late (**c**) induced *Galc-iKOs*. (**b, d**) GALC activity substantially returned in the sciatic nerves (12-14%) and slightly in livers (5-9%), but not at all in spleens (3-7%) of early and late induced moribund *Galc-iKO* mice. n=4. The experiment was repeated three times with samples from different animals. All data are presented as mean values +/- SD.

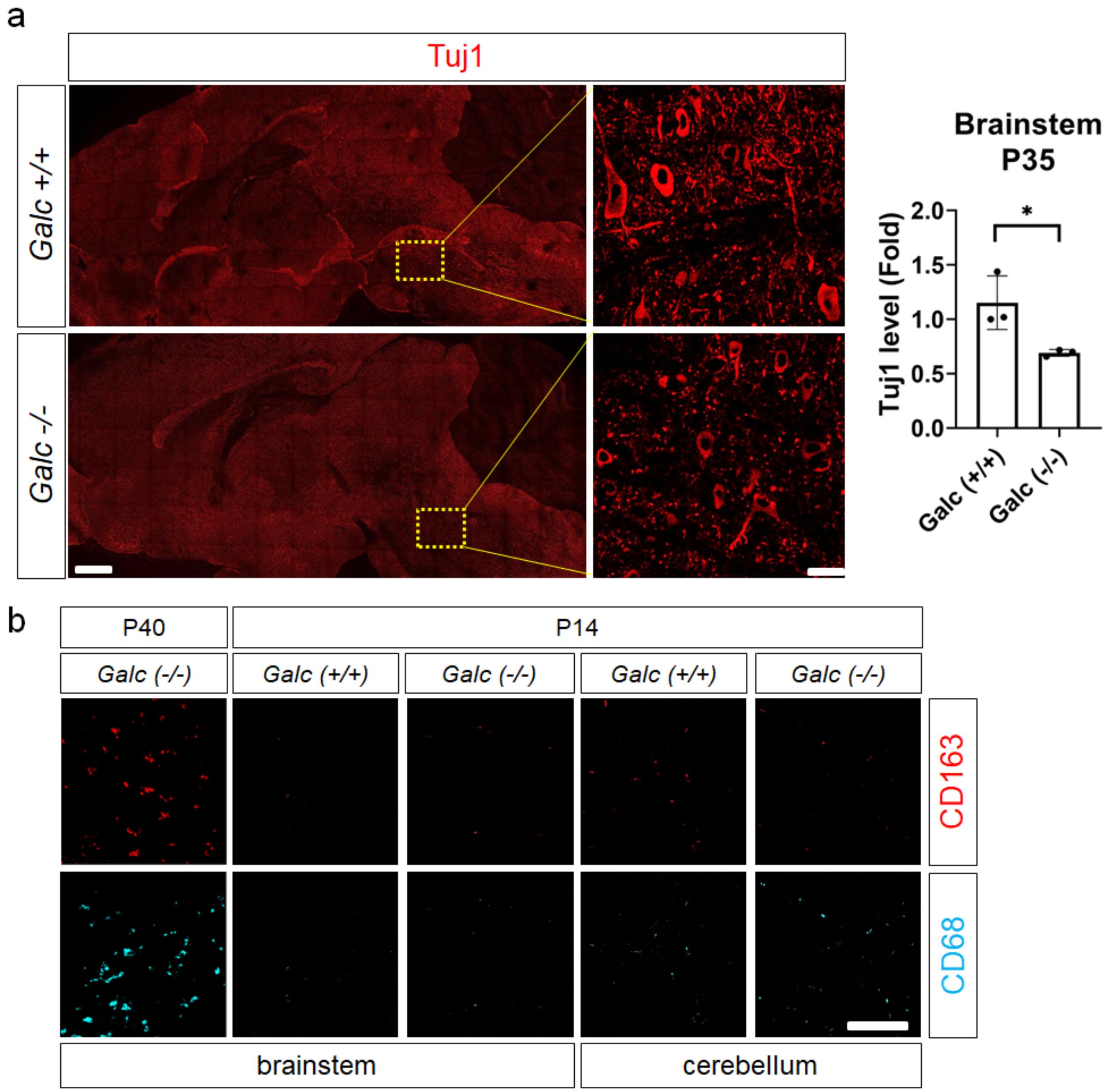

Figure S3. Additional immunohistochemical analyses of *Galc-KO* brainstems in the progression of the disease. (**a**) Neuron-specific class III beta-tubulin, Tuj1, was significantly reduced in the brainstem of moribund P35 *Galc-KO* mice, compared to WT. Tuj1 level was quantified with integrated density that is the product of positive area and mean intensity of the signal, after subtracting background value from the another section stains devoid of the primary antibody. Unpaired two-tailed Student's t-test was used. n=3 per genotype. All data are presented as mean values +/- SD. **p*=0.0326. Scale bar=2 mm (whole brain) and 50 μm (inset). (**b**) CD68 (red) and CD163 (cyan) positive microglia are not

activated in the brainstem and cerebellum of P14 *Galc*-KO, but are highly increased in the moribund P40 KO brains. Scale bar=50 μm.

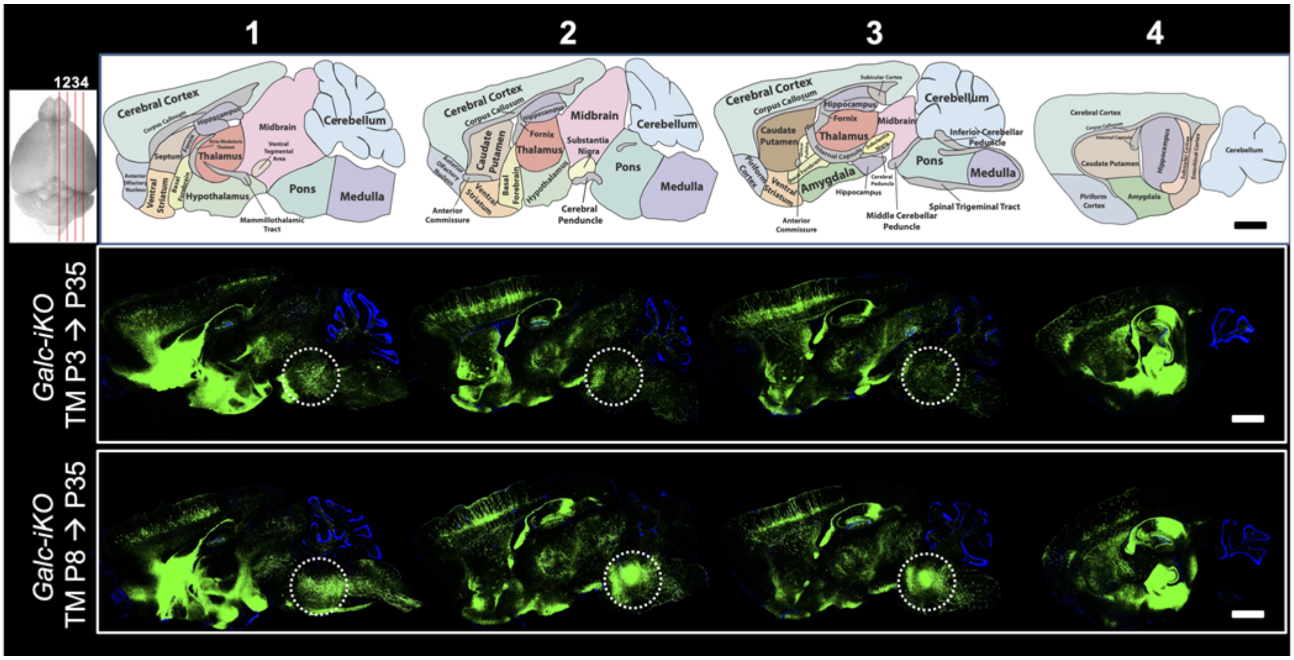

Figure S4. Representative serial sagittal brain sections of *Galc-iKO*;Thy1.1-YFP. Imaging of different sagittal planes covering most whole brains at P35 shows a more robust decrease of YFP signals in the brainstem (white dotted circles) of the *Galc-iKO* induced at P3, compared to the induction at P8. n=3 per genotype. Scale bars=2 mm. The anatomical cartoons of the mouse brain were drawn by co-author, Jacob Favret.

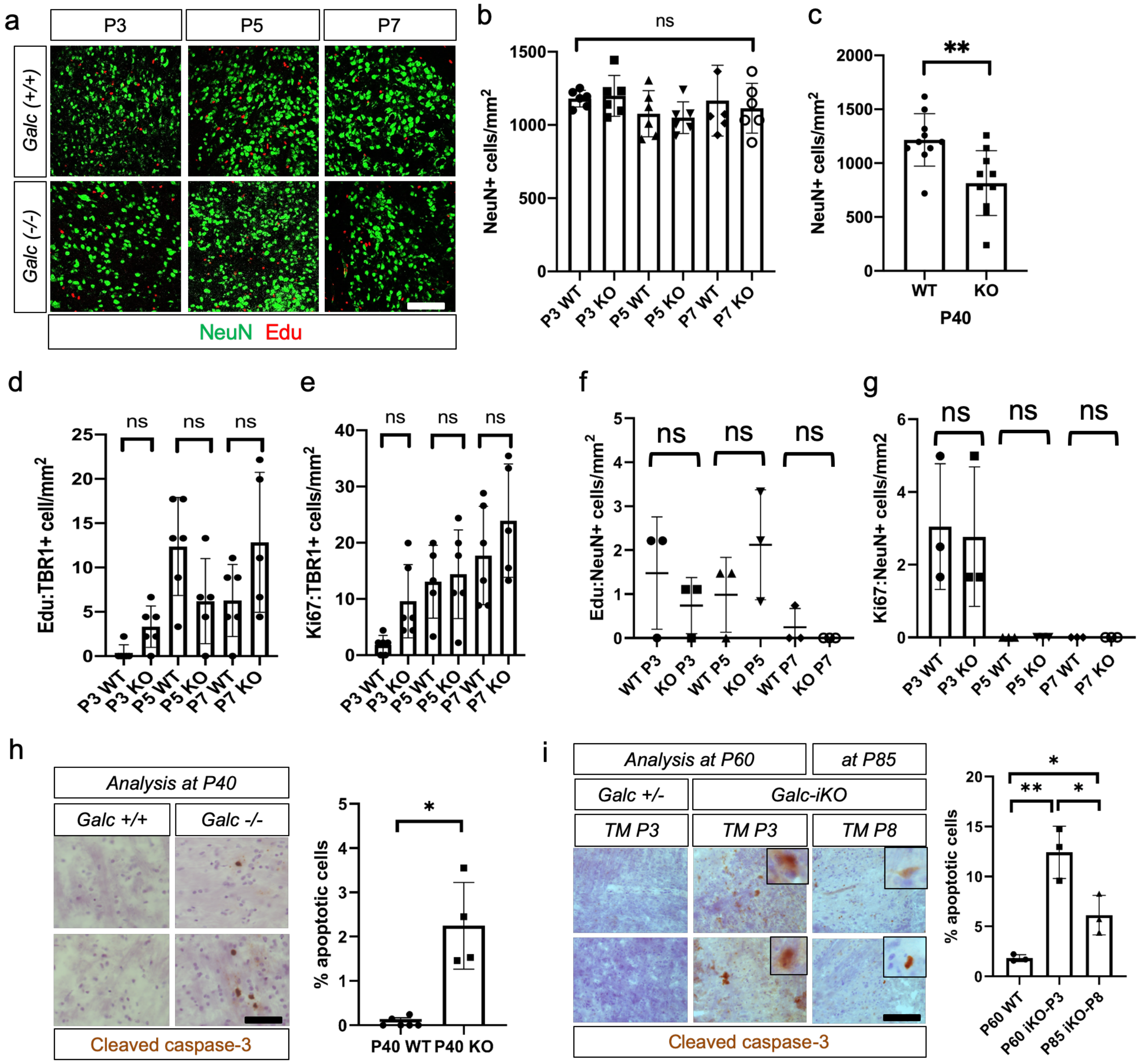

Figure S5. The moribund GALC-depleted brainstem had reduced NeuN+ neurons. Immunostaining (**a**) and counting of NeuN+ neurons (**b**) shows that there were not changes in P3, 5, and 7 brainstems of *Galc*-KO. n=6. Scale bar=50 μm. (**c**) The end-stage KO brainstem has a significant reduction of NeuN+ neurons. n=9. $**p=0.0041$. Proliferating TBR1+ (**d, e**) or NeuN+ (**f, g**) neurons were not significantly changed by GALC deficiency during the P3-7 period. Edu was administrated 24 hours before of the analysis. n=6 (**d, e**) and n=3 (**f, g**). Immunostaining of cleaved caspase-3 revealed that apoptotic cell death per total cell (nuclei number) is significantly increased in the brainstem of moribund *Galc*-KO (**h**) and *Galc*-iKOs (**i**). *Galc*-iKO≤P4 had more prominent apoptotic signals than *Galc*-iKO≥P6. Scale bar=100 μm. n=6 for WT and n=4 for KO in **h**, and n=3 in **i**. P values are 0.0206 (h), 0.0023 (i, WT vs iKO-P3), 0.0205 (i, WT vs iKO-P8), and 0.0328 (i, iKO P3 vs P8). All data are presented as mean values +/- SEM. Two-way ANOVA with Tukey's multiple comparison

tests (**b, d-g**) and unpaired two-tailed Student's t-test (**c, h, i**) were used. *p < 0.05 and **p < 0.01. ns; not significant.

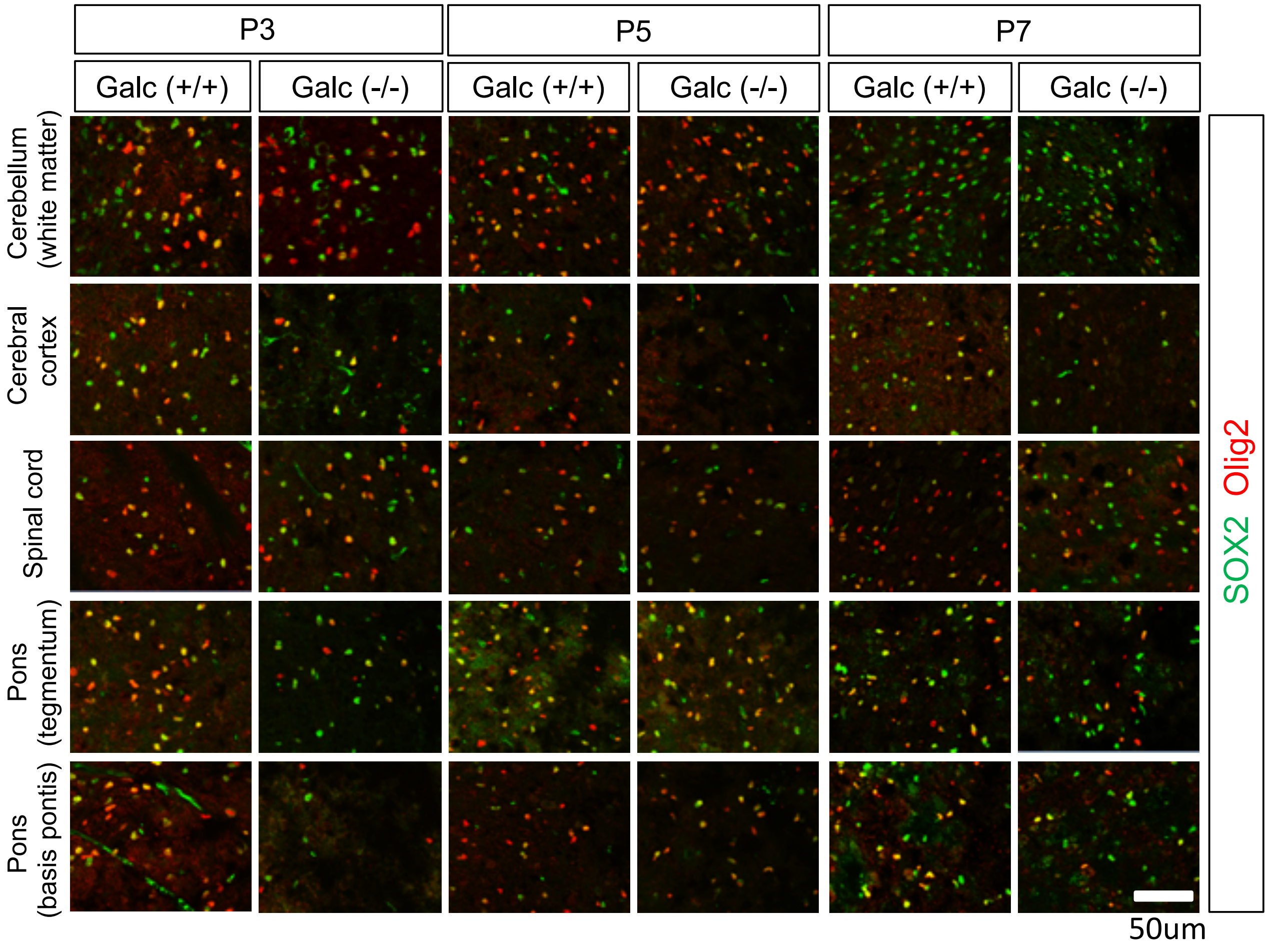

Figure S6. Representative images of SOX2 (green) and Olig2 (red) staining in the brains of *Galc*-WT and KO at P3, P5 and P7. The experiment was repeated three times with samples from different animals.

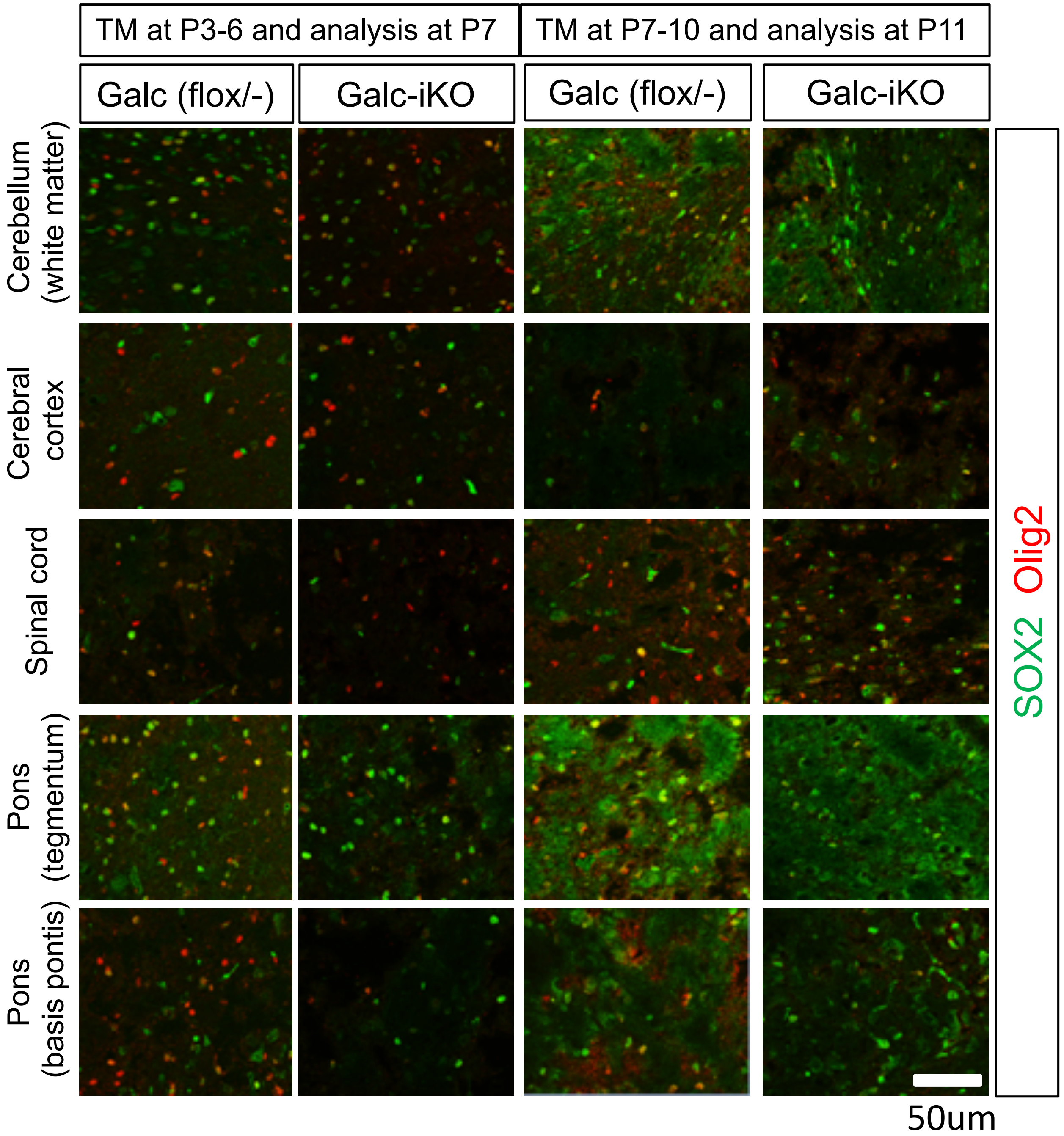

Figure S7. Representative images of SOX2 (green) and Olig2 (red) staining in the brains of *Galc*-iKO mice 24 hours after induction starting at either P3 or P7. The experiment was repeated three times with samples from different animals.

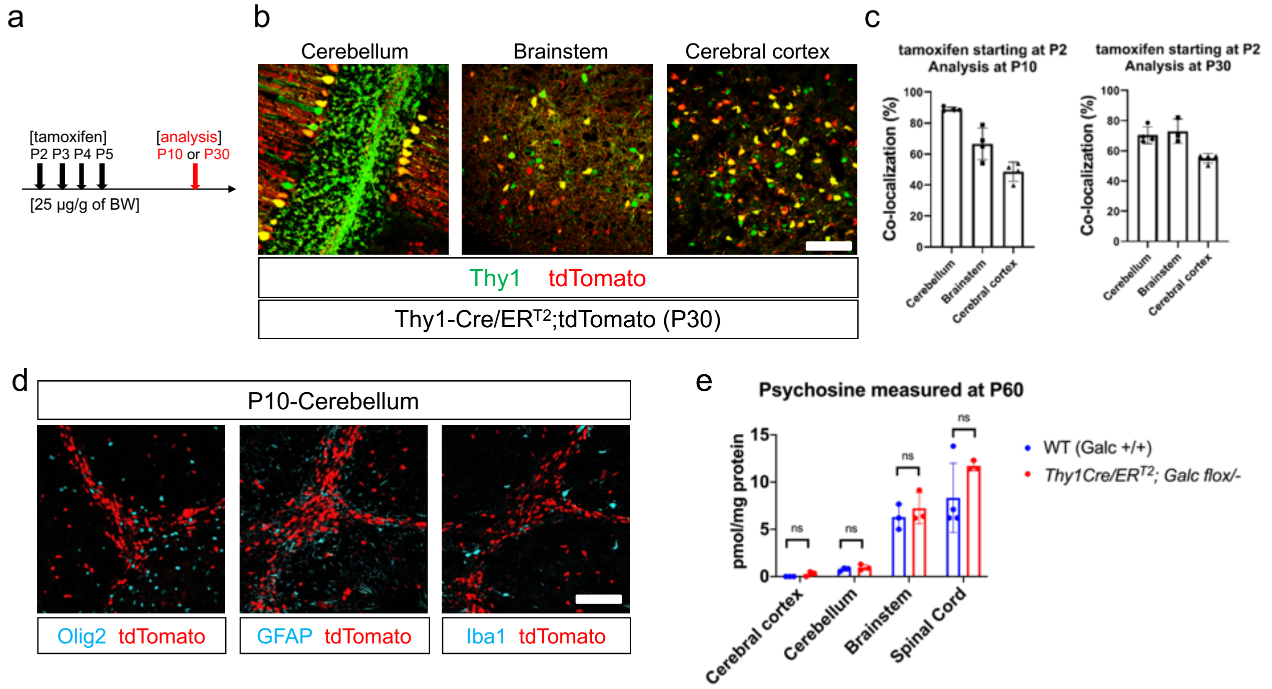

Figure S8. Thy1-Cre/ER^{T2} transgenes specifically undergo recombination in neurons. Tamoxifen was injected into Thy1-Cre/ER^{T2}; tdTomato starting at P2, and the mice were dissected at P10 or P30 (**a**). Analysis of major sub-brain regions such as cerebellum, brainstem and cerebral cortex (**b**) showed that more than 65% of Thy1-positive cells overlap with the tdTomato in the hindbrain. The co-localization rate was about 50% in the cerebral cortex (**c**). The tdTomato signals were not overlapped with other cell markers, Olig2, GFAP and Iba1, indicating neuron-specific recombination (**d**). Scale bar=50 μm. n=3 per genotype. (**e**) Psychosine levels were measured in the cerebral cortex, cerebellum, brainstem, and spinal cord of P60 Thy1-Cre/ER^{T2} driven *Galc*-CKO induced at P3. Their levels were not different to WT, indicating the observed brainstem neuronal maturation defect was likely not psychosine-dependent. n=3 except WT spinal cord that is n=4. Two-way ANOVA with Tukey's multiple comparison test was performed. ns; not significant. Data are presented as mean values +/- SD.
