# Supplementary material for: Brainstem development requires galactosylceramidase and is critical for pathogenesis in a model of Krabbe disease
Source: Nat Commun. 2020 Oct 23;11:5356. doi: 10.1038/s41467-020-19179-w (PMC7584660; doi:10.1038/s41467-020-19179-w)
Supplement: Supplementary file 3 — Reporting Summary [file 41467_2020_19179_MOESM3_ESM.pdf]

nature research

Corresponding author(s): Daesung Shin

Last updated by author(s): Sep 23, 2020

Reporting Summary

Nature Research wishes to improve the reproducibility of the work that we publish. This form provides structure for consistency and transparency in reporting. For further information on Nature Research policies, see our Editorial Policies and the Editorial Policy Checklist.

Statistics

For all statistical analyses, confirm that the following items are present in the figure legend, table legend, main text, or Methods section.

n/a	Confirmed	
☐	☒	The exact sample size (*n*) for each experimental group/condition, given as a discrete number and unit of measurement
☐	☒	A statement on whether measurements were taken from distinct samples or whether the same sample was measured repeatedly
☐	☒	The statistical test(s) used AND whether they are one- or two-sided<br>*Only common tests should be described solely by name; describe more complex techniques in the Methods section.*
☒	☐	A description of all covariates tested
☒	☐	A description of any assumptions or corrections, such as tests of normality and adjustment for multiple comparisons
☐	☒	A full description of the statistical parameters including central tendency (e.g. means) or other basic estimates (e.g. regression coefficient) AND variation (e.g. standard deviation) or associated estimates of uncertainty (e.g. confidence intervals)
☐	☒	For null hypothesis testing, the test statistic (e.g. *F*, *t*, *r*) with confidence intervals, effect sizes, degrees of freedom and *P* value noted<br>*Give P values as exact values whenever suitable.*
☒	☐	For Bayesian analysis, information on the choice of priors and Markov chain Monte Carlo settings
☒	☐	For hierarchical and complex designs, identification of the appropriate level for tests and full reporting of outcomes
☒	☐	Estimates of effect sizes (e.g. Cohen's *d*, Pearson's *r*), indicating how they were calculated

Our web collection on statistics for biologists contains articles on many of the points above.

Software and code

Policy information about availability of computer code

Data collection	All immunohistochemical images were acquired using Lecia SP5 laser-scanning confocal microscopic analysis (Leica Biosystems). Raw data for GALC activity and protein quantification for tissue lysates were obtained with microplate reader (BioTek Cytation 5MV). Quantitative RT-PCR data were obtained with CFX96/384 RealTime PCR machine (Bio-Rad), and all other PCR reactions including genotyping were performed using Applied Biosystems 96-well thermal cycler. Western blot and Northern blot images were obtained with Odyssey (LI-COR) and Kodak X-ray film developer. For the images of transmission electron microscopy, epon-embedded tissue block was processed with ultratome (Leica) and the picture was taken with a Tecnai electron microscope. Rota-rod analysis was performed by using UGO Basile Rota-rod.
Data analysis	Immunofluorescence images were analyzed using ImageJ (NIH, ver. 2.0.0-rc-69/1.52i) and ilastik (ver. 1.4b3). All statistics were analyzed with Graphpad Prism (ver. 8).

For manuscripts utilizing custom algorithms or software that are central to the research but not yet described in published literature, software must be made available to editors and reviewers. We strongly encourage code deposition in a community repository (e.g. GitHub). See the Nature Research guidelines for submitting code & software for further information.

Data

Policy information about availability of data

All manuscripts must include a data availability statement. This statement should provide the following information, where applicable:

- Accession codes, unique identifiers, or web links for publicly available datasets
- A list of figures that have associated raw data
- A description of any restrictions on data availability

The raw data underlying Figures 1-10 and Supplementary Figures 2, 3, 5 and 8 are available via a source data file submitted with this manuscript. All other data are available from the corresponding authors upon reasonable request.

April 2020

Field-specific reporting

Please select the one below that is the best fit for your research. If you are not sure, read the appropriate sections before making your selection.

- [x] Life sciences
- [ ] Behavioural & social sciences
- [ ] Ecological, evolutionary & environmental sciences

For a reference copy of the document with all sections, see nature.com/documents/nr-reporting-summary-flat.pdf

Life sciences study design

All studies must disclose on these points even when the disclosure is negative.

Sample size	We chose the sample size (at least three biological replicates, i.e., mice or cell culture preparations) based upon historical practices, in line with recent relevant publications, for instance Li et al. Genetic ablation of acid ceramidase in Krabbe disease confirms the psychosine hypothesis and identifies a new therapeutic target, PNAS 116 (40) 20097-20103 (2019), as well as Marshall et al. Long-Term Improvement of Neurological Signs and Metabolic Dysfunction in a Mouse Model of Krabbe's Disease after Global Gene Therapy. Mol Ther. 26(3): 874–889 (2018). The sample sizes are provided for each experiment in the respective figures by showing individual symbols for each biological replicate or by indicating the number of replicates in the legends.
Data exclusions	No data was excluded in this study.
Replication	All experiments were replicated at least 3 times with similar results that was mentioned in the figure legends.
Randomization	Sections used for imaging were selected randomly. Animals are randomized and were compared to littermates.
Blinding	No blinding was done but experiments were carried out and analyzed by at least two different operators.

Reporting for specific materials, systems and methods

We require information from authors about some types of materials, experimental systems and methods used in many studies. Here, indicate whether each material, system or method listed is relevant to your study. If you are not sure if a list item applies to your research, read the appropriate section before selecting a response.

Materials & experimental systems

n/a	Involved in the study	
[ ]	[x]	Antibodies
[ ]	[x]	Eukaryotic cell lines
[x]	[ ]	Palaeontology and archaeology
[ ]	[x]	Animals and other organisms
[x]	[ ]	Human research participants
[x]	[ ]	Clinical data
[x]	[ ]	Dual use research of concern

Methods

n/a	Involved in the study	
[x]	[ ]	ChIP-seq
[x]	[ ]	Flow cytometry
[x]	[ ]	MRI-based neuroimaging

Antibodies

Antibodies used

Rabbit anti-GFAP, Abcam Cat#ab7260, RRID:AB_305808
Mouse anti-TLR2, R and D Systems Cat# AF1530, RRID:AB_354847
Rat anti-CD68, Bio-Rad Cat# MCA1957, RRID:AB_322219
Mouse anti-CD163, Bio-Rad Cat# MCA342GA, RRID:AB_2074558
Rabbit anti-tdTomato, Origene Cat#TA150128, RRID:AB_2571847
Rabbit anti-beta-tubulin, Novus Cat# NB600-936, RRID:AB_10000656
Mouse anti-NeuN, Millipore Cat# MAB377, RRID:AB_2298772
Mouse anti-Tuj1, Covance Cat# MMS-435P, RRID:AB_2313773
Rat anti-HA High Affinity, Roche Cat# 11867423001, RRID:AB_390918
Rabbit anti-Olig2, Proteintech Cat# 13999-1-AP, RRID:AB_2157541
Rabbit anti-TBR1, Abcam Cat# ab31940, RRID:AB_2200219
Mouse anti-Sox2, R and D Systems Cat# MAB2018 RRID:n/a
Rat anti-Ki67, Thermo Fisher Scientific Cat# 14-5698-80, RRID:AB_10853185
Rabbit anti-Cleaved Caspase-3, Cell Signaling Technology Cat# 9664, RRID:AB_2070042
Chicken anti-GALC, CL1021AP A gift from Chris Lee and Christopher Eckman (Lee et al. J Neurosci 2010)
Rabbit anti-IBA1 (for immunocytochemistry) Wako Chemicals Cat# 019-19741 RRID:AB_839504
Rabbit anti-human OLIG2 Proteintech Proteintech Cat# 13999-1-AP, RRID:AB_2157541
Alexa Fluor 488-AffiniPure F(ab')2 Fragment Donkey anti-Chicken IgY, Jackson ImmunoResearch Labs Cat# 703-546-155, RRID:AB_2340376

	Rhodamine Red-X-AffiniPure Donkey Anti-Rabbit IgG, Jackson ImmunoResearch Labs Cat# 711-295-152, RRID:AB_2340613<br>Alexa Fluor 647 AffiniPure F(ab')$_2$ Fragment Donkey Anti-Rabbit IgG, Jackson ImmunoResearch Labs Cat# 711-606-152, RRID:AB_2340625<br>Goat anti-Mouse IgG2b Cross-Adsorbed Alexa Fluor 594, Thermo Fisher Scientific Cat# A-21145, RRID:AB_2535781<br>Alexa Fluor 594-AffiniPure Goat Anti-Mouse IgG, Fc subclass 2a, Jackson ImmunoResearch Labs Cat# 115-585-206, RRID:AB_2338886<br>Cy3-AffiniPure Donkey Anti-Goat IgG (H+L), Jackson ImmunoResearch Labs Cat# 705-165-003, RRID:AB_2340411<br>Alexa Fluor 488-AffiniPure Donkey Anti-Rat IgG, Jackson ImmunoResearch Labs Cat# 712-545-153, RRID:AB_2340684<br>Rhodamine (TRITC)-AffiniPure Donkey Anti-Rat IgG, Jackson ImmunoResearch Labs Cat# 712-025-153, RRID:AB_2340636<br>Donkey Anti-Rabbit Rabbit IgG (H+L) Polyclonal, HRP-Conjugated, Novus Cat# NB 7185, RRID:AB_524677<br>Peroxidase-AffiniPure Donkey Anti-Chicken IgY (IgG), Jackson ImmunoResearch Labs Cat# 703-035-155, RRID:AB_10015283<br>Peroxidase-AffiniPure Goat Anti-Rat IgG, Light Chain Specific, Jackson ImmunoResearch Labs Cat# 112-035-175, RRID:AB_2338140
Validation	Anti-GALC antibody was validated on GALC-KO brain tissues, where its signal was completely absent, while it was positive in WT tissues. In addition, the specificity of this antibody was amply validated by the original paper that generated it (Lee et al., J Neurosci 2010) and our recent paper (Weinstock et al, Neuron 2020). All commercially available antibodies used in-vivo, for western blot or for immunohistochemistry were validated for their application by manufacturers.

Eukaryotic cell lines

Policy information about cell lines

Cell line source(s)	HEK293T cells (human, ATCC CRL-1573), ES cells from 129S6 (Roswell Park Cancer Institute)
Authentication	None of the cell lines have been authenticated.
Mycoplasma contamination	The cell line had a negative result from routine mycoplasma contamination test (PCR).
Commonly misidentified lines<br>(See ICLAC register)	None of the cell used in this study are listed in the ICLAC database.

Animals and other organisms

Policy information about studies involving animals; ARRIVE guidelines recommended for reporting animal research

Laboratory animals	Galc-flox/+ and Galc+/- were generated in this study. All animals were maintained on the congenic background of C57BL/6N, which breeder were purchased from Charles River (Wilmington, MA). CAG-Cre/ERT(JAX#004682), CMV-Cre (JAX#006054), Del-FLPe (JAX#012930), tdTomato (JAX#007905), Thy1.1-YFP (JAX#003782), and Thy1-Cre/ERT2 (JAX#012708) were purchased from The Jackson laboratory (Bar Harbor, ME). Both males and females were used. Animals of age P0-85 were used.
Wild animals	No wild animals were used in this study.
Field-collected samples	No field-collected samples were used in this study.
Ethics oversight	The mouse work was performed under the study protocol UB1254M and UB1188M, as approved by the Institutional Animal Care and Use Committee (IACUC) of University at Buffalo (SUNY) and Roswell Park Cancer Institute (RPCI).

Note that full information on the approval of the study protocol must also be provided in the manuscript.
